# The Preventive Effect of Zuogui Wan on Offspring Rats' Impaired Glucose Tolerance Whose Mothers Had Gestational Diabetes Mellitus

**DOI:** 10.1155/2016/9417362

**Published:** 2016-02-29

**Authors:** Yuwei Wang, Qianjin Feng, Xin Niu, Kaixia Xu, Yingli Wang, Jinlong Wang, Qiuju Li, Yingqiu Mao, Shuangrong Gao

**Affiliations:** ^1^Beijing University of Chinese Medicine, No. 11 Beisanhuan Donglu, Chaoyang District, Beijing 100029, China; ^2^Shanxi University of Traditional Chinese Medicine, No. 121 Daxue Street, Gaoxiaoyuan District, Taiyuan, Shanxi 030619, China; ^3^Zhejiang University, Hangzhou, Zhejiang 310058, China; ^4^Institute of Chinese Materia Medica, China Academy of Chinese Medical Sciences, Beijing 100700, China

## Abstract

In this experiment, we used streptozotocin (STZ) to establish a model of gestational diabetes mellitus (GDM) rats, where Zuogui Wan was given to GDM rats. After pregnancy, offspring rats were divided into 4 groups: control group, high fat and sugar as the control group, GDM group, and Zuogui Wan GDM group. Rats in high fat and sugar as the control group, GDM group, and Zuogui Wan GDM group were fed with high fat and sugar diet. Rats in control group were fed the basic diet. The means of 2hPG were higher than 7.8 mmol·L^−1^ and lower than 11.1 mmol·L^−1^ on the rats of GDM group on week 15, and IGT models were successful. Body weight, abdominal fat weight, the ratio of abdominal fat weight and body weight, fasting plasma glucose, 2hPG, insulin, leptin, total cholesterol, and low density lipoprotein (LDL) of Zuogui Wan GDM group were significantly lower than GDM group. The level of adiponectin in Zuogui Wan GDM group was significantly higher than GDM group. And we concluded that giving Zuogui Wan to GDM rats can have a preventive effect on the offsprings' IGT induced by high fat and sugar diet.

## 1. Introduction

In the 1990s, British professor Barker [1] put forward “the fetal origins of adult disease” hypothesis. The hypothesis proposed that cardiovascular disease, hypertension, abnormal glucose metabolism, and other chronic diseases are caused by an adverse environment for the early fetus. Specifically, an adverse intrauterine environment prompts the early fetus to make adaptive adjustments to its own metabolism and the organizational structure of organogenesis. This adaptation will lead to permanent changes in tissue structure and function, which will then lead to adult diseases. From then on, the study of the origin of health and disease has become a worldwide research interest [2–5]. Traditional Chinese medicine theories in Huangdi Neijing believe that “kidney essence is stored in the Kidney,” and “the kidney is responsible for birth, growth and reproduction.” The kidney is the inborn origin of the body. If kidney function is poor in the mother, then the offspring will likely develop many diseases when growing up. We believe that the theory of traditional Chinese medicine is consistent with “the fetal origins of adult disease” hypothesis. Zuogui Wan is a prescription from a Chinese medicine book “Jingyue Quanshu,” whose effect is to nourish Yin and tonify the kidney. There are 8 ingredients in Zuogui Wan: prepared rhizome of adhesive* Rehmannia*, Rhizoma Dioscoreae, Barbary Wolfberry fruit,* Cornus officinalis*, China dodder, Colla Cornus Cervi, tortoise shell glue, and medicinal* Cyathula* root. In this experiment we gave Zuogui Wan to GDM rats and supplemented the inborn origin of the body in fetus to GDM rats. The results demonstrated that Zuogui Wan can prevent IGT induced by high fat and sugar diet in offspring rats.

## 2. Materials

### 2.1. Experimental Animals

Experimental Wistar rats (female = 100, male = 100) were all adult rats with a body weight between 200 g and 250 g. The experimental animals were provided by Beijing Vital River Laboratory Animal Technology Co., Ltd. (license number: SCXK (BJ) 2012-0001). The conduct of the experiments was in accordance with international zoology ethical standards.

### 2.2. Medicines and Reagent

Zuogui Wan is a prescription from a Chinese medicine book “Jingyue Quanshu,” made of prepared rhizome of adhesive* Rehmannia*, Rhizoma Dioscoreae, Barbary Wolfberry fruit,* Cornus officinalis*, China dodder, Colla Cornus Cervi, tortoise shell glue, and medicinal* Cyathula* root with a 8 : 4 : 4 : 4 : 4 : 4 : 4 : 3 ratio. Ingredients were purchased from Beijing Tong Ren Tang and then verified by the Department of Medicine of Beijing University of Chinese Medicine as genuine. We used ceramics to decoct and extract Chinese herbal medicines. The mass concentration was 1 g·mL^−1^ of dried herbs. Streptozotocin (STZ) was produced by American Sigma Corporation (batch: B64927). We adjusted the STZ solution to acidity (pH = 4.2) by 0.1 mol·L^−1^ citric acid buffer solution, which was purchased from Beijing fraternity Port Company. Uric sugar test paper was purchased from Uritest Guilin Medical Electronic Sales Co., Ltd. (batch: 56130184). Chloral hydrate was purchased from Tianjin Fucheng Chemical Reagent Factory. Triglyceride detection kit and cholesterol detection kit were purchased from Beijing Wan Tai Derui Diagnostic Technology Co., Ltd. (batch: ZL2103AA02T and batch: ZL2105AA31).

### 2.3. Feed

A basic diet (ID = 1022) and high fat and sugar diet (protein 20%, carbohydrate 20%, and fat 60%) were all purchased from Beijing, China Huafukang Biological Technology Co., Ltd. (license number: SCXK (BJ) 2009-0008).

### 2.4. Instruments

Instruments used in this study included blood sugar detector (Johnson stable fold easily type LF033/V02), glucose test strips (Johnson Lot3354358), automatic biochemical analyzer (Toshiba TBA-40PR), −80°C ultralow temperature refrigerator (Plus Value, Thermo, USA), 10 mL disposable venous blood collection (Hunan Sanli Industrial Co., Ltd.), and 100 *μ*L–1000 *μ*L micro sample adding device (Dragonlab Company, USA).

## 3. Methods

### 3.1. Model Preparation

#### 3.1.1. Model Preparation for Pregnant Rats

100 female rats were fed for one week under the following conditions: 20–22°C, 30%~65% for relative humidity, 150~300 Lx for illumination, and 12 : 12 day and night ratio. During the adaptive feeding period, saline (20 mL·kg^−1^) was administered to rats by gavage once a day. One week later, 100 sets of rat cages were fixed in shelves of hanging type. Meanwhile, a tray was placed under each rat cage. 100 female and 100 male rats were put together in cages in which the female/male ratios were 1 : 1. At the same time, two methods were used to determine pregnancy of female rats. Firstly, after 12 hours, the method of pessary in tray was used to observe whether there were pessaries (ivory and solid jelly). Then, rats with pessary in tray were taken to do vaginal smears. If sperm had been found by microscopic examination [4], then they were labeled as pregnant rats (0 d). A total of 34 pregnant rats were detected in this experiment. Then, the pregnant rats were taken out; 23 were fed with high fat and sugar diet, and 11 were fed with basic diet.

#### 3.1.2. Establishment of Model of Rats of GDM

The 34 pregnant rats taken from the preparation stage (the first day of pregnancy) were fasted for 12 hours. After that, 23 of them were injected peritoneally with dissolved STZ (40 mg/kg) and labelled as Zuogui Wan GDM group (*n* = 12) and GDM group (*n* = 11) and the remaining 11 rats were labelled as normal pregnant group and received an injection of sodium citrate buffer solution. The 23 rats were given normal water and high fat and sugar diet after 4 hours of giving STZ. The film forming standard of gestational diabetes was fasting blood glucose after 72 hours of the injection of STZ ≥ 11.1 mmol/L or random blood glucose ≥ 16.7 mmol/L and urine glucose > ++. Rats of 4-day pregnancy in Zuogui Wan GDM group and GDM group were all in accordance with film forming standard of GDM [6].

### 3.2. Grouping and Treatment

#### 3.2.1. Grouping and Treatment on Mother Rats

The 34 pregnant rats were divided into three groups: Zuogui Wan GDM group (*n* = 12), GDM group (*n* = 11), and normal pregnancy group (*n* = 11). Rats in both Zuogui Wan GDM group and GDM group were fed with high fat and sugar diet. Rats in normal pregnancy group were fed basic diet. Zuogui Wan decoction (i.g., 1 g·mL^−1^, 20 mL·kg^−1^) was administered once a day to rats in Zuogui Wan GDM group by gavage for 19 days. Saline (20 mL·kg^−1^) was administered by gavage once a day to rats in GDM group and normal pregnancy group for 19 days.

#### 3.2.2. Grouping and Treatment on Offspring Rats

**Figure 1:**
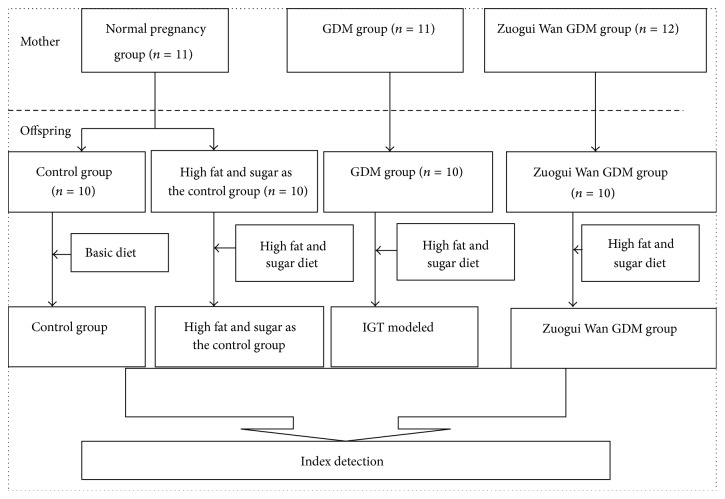
Grouping on mother rats and offspring rats.

20 to 22 days after pregnancy, the pregnant rats gave birth to the first-generation rats (offspring for short). As can be seen from Figure 1, after 21 days of breastfeeding, one male newborn rat was selected randomly from every brood of Zuogui Wan GDM group and GDM group, and the names of these two groups were unchanged (one brood of offspring in GDM group did not have any males, and two broods of offspring in Zuogui Wan GDM group did not have any males). Two male newborn rats were randomly selected from every brood of normal pregnancy group (one brood of offspring control group did not have any males); the two groups were named as control group and high fat and sugar as the control group. At the age of 4 weeks, each offspring group was treated with basic feed, ad libitum food and water. At the age of 5 weeks, each group was treated as follows: rats in GDM group (*n* = 10) were fed with high fat and sugar diet, ad libitum and water; rats in Zuogui Wan GDM group (*n* = 10) were fed with high fat and sugar diet, ad libitum food and water; rats in high fat and sugar as the control group (*n* = 10) were fed with high fat and sugar diet, ad libitum food and water; rats in control group (*n* = 10) were fed the basic diet, ad libitum food and water.

From week 8 on, fasting blood glucose and 2-hour plasma glucose (2hPG for short) were detected and recorded every week in each group. The means of 2hPG were higher than 7.8 mmol·L^−1^ and lower than 11.1 mmol·L^−1^ on the rats of GDM group on week 15, and IGT models were successful. One rat in control group was failed when taking blood.

### 3.3. Detection Index

#### 3.3.1. Body Weight

Body weight was measured after the offspring were born (the total weight of the brood divided by the number), after 3 weeks of breastfeeding, and in week 6, week 8, week 10, week 12, week 14, and week 15 of all rats which formed new groups.

#### 3.3.2. Fasting Blood Glucose and 2hPG

From week 8 on, fasting blood glucose and 2hPG were measured every week in each group.

#### 3.3.3. Weight of Fat

In week 15, the IGT models were successful in GDM group, abdominal fat (which mainly contains the peritoneum fat, testis fat pad, and bilateral perirenal fat) was taken out, the total weight was checked by electronic balance, and the ratio of abdominal fat and body weight was calculated.

#### 3.3.4. Total Cholesterol, Triglyceride, Low Density Lipoprotein, and High Density Lipoprotein

Blood was drawn from the abdominal artery from all rats. All the blood samples were centrifuged to get serum. Total cholesterol, triglyceride, low density lipoprotein, and high density lipoprotein were detected by using enzyme-conjugated colorimetric analysis method by automatic biochemistry analyzer according to the procedures of kits.

#### 3.3.5. Insulin, Leptin, Adiponectin, and Insulin Resistance Index

Blood was drawn from the abdominal artery from all rats. All the blood samples were centrifuged to get serum. The insulin level was detected by using enzyme-immunoassay method according to the procedures of kits: insulin resistance index (HOMA-IR) = FINS*∗*FPG/22.5.

### 3.4. Data Processing

Data was processed in SPSS18.0 statistical software. Differences between groups were compared with one-way analysis of variance. *P* < 0.05 represented statistical significance in this study. The comparison results were shown by notations as follows: ^*∗*^ *P* < 0.05; ^*∗∗*^ *P* < 0.01; ^*∗∗∗*^ *P* < 0.001.

## 4. Results

### 4.1. Body Weight

**Figure 2:**
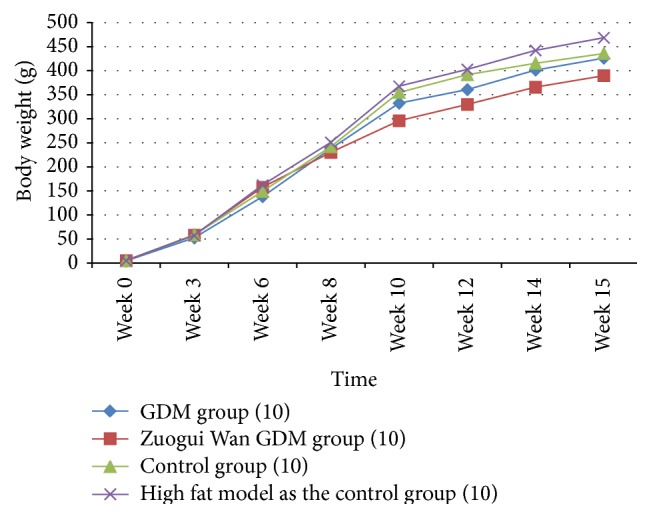
Change of body weight.

As can be seen from Table 1 and Figure 2, compared with GDM group, body weight in week 0 was significantly higher (*P* < 0.05, *P* < 0.01, and *P* < 0.01) in Zuogui Wan gestational diabetes group, control group, and high fat model as the control group. In week 3, body weight was significantly higher (*P* < 0.01) in Zuogui Wan GDM group, control group, and high fat model as the control group compared with GDM group. In week 6, compared with GDM group, body weight was significantly higher (*P* < 0.01, *P* < 0.001) in Zuogui Wan GDM group and high fat model as the control group; the means of body weight in control group was higher than GDM group, but the difference was not significant. From week 8 to week 12, the means of body weight in GDM group was higher than that in Zuogui Wan GDM group, but the difference was not significant. From week 8 to week 10, body weight in control group and high fat model as the control group was higher than GDM group, but the difference was not significant. In week 12, compared with GDM group, body weight in high fat model as the control group was significantly higher (*P* < 0.05), body weight in control group was higher than that in GDM group, but the difference was not significant. In week 14 and week 15, compared with GDM group, body weight in Zuogui Wan GDM group was significantly lower (*P* < 0.05), body weight in high fat model as the control group was significantly higher (*P* < 0.05), and body weight in control group was higher, but the difference was not significant.

### 4.2. Fasting Blood Glucose and 2hPG

**Figure 3:**
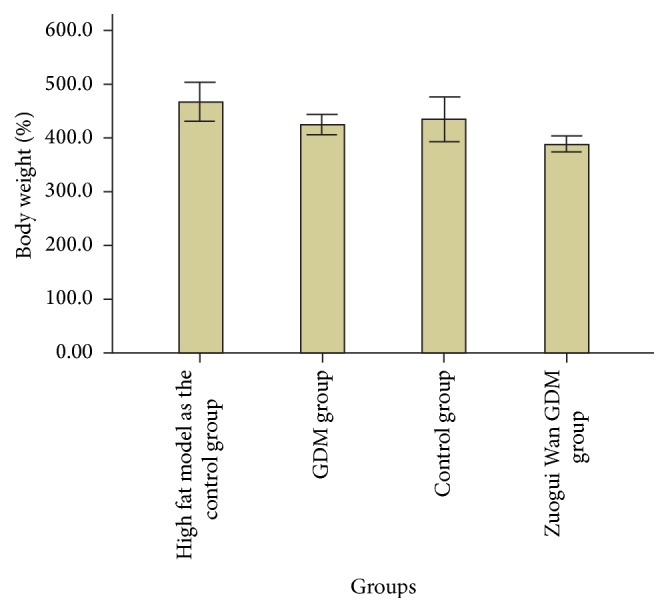
Body weight in week 15.

**Figure 4:**
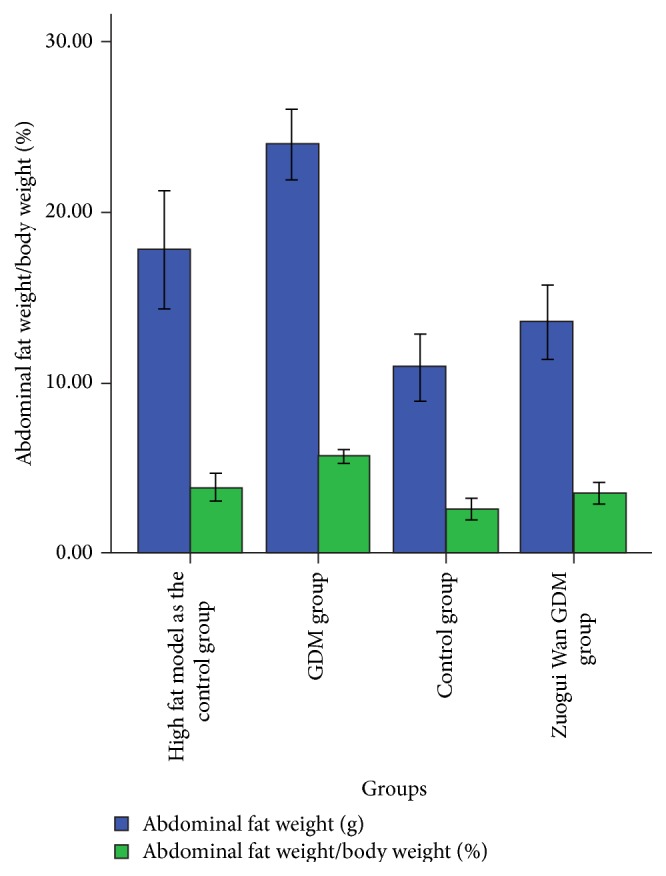
Abdominal fat weight and abdominal fat weight/body weight.

As can be seen from Table 2 and Figures 3 and 4, compared with GDM group, abdominal fat weight and abdominal fat weight/body weight in Zuogui Wan GDM group, control group, and high fat model as the control group were significantly lower (*P* < 0.001).

### 4.3. Fasting Blood Glucose and 2hPG

**Figure 5:**
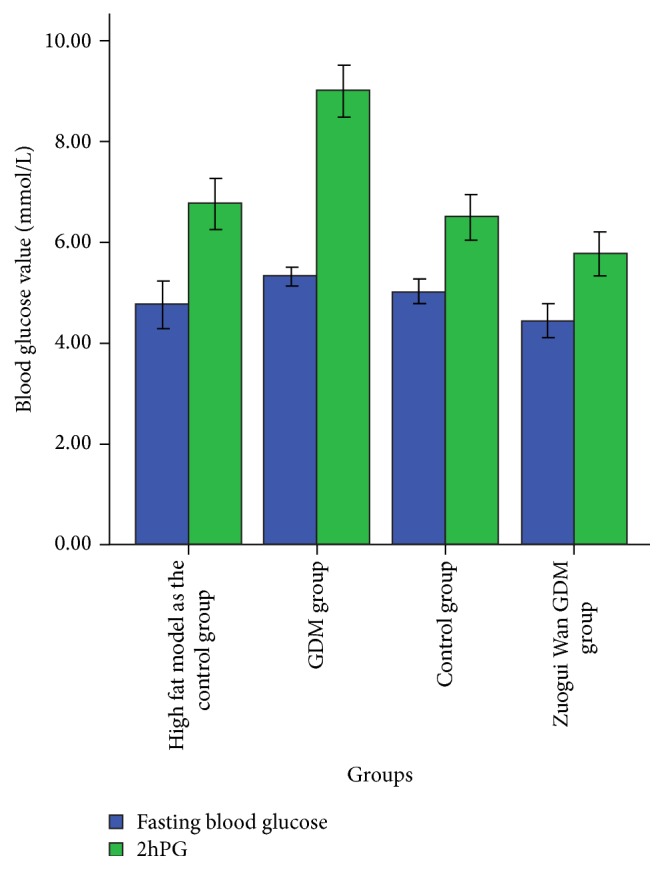
Fasting blood glucose and 2hPG.

It is apparent in Table 3 and Figure 5 that, compared with GDM group, fasting blood glucose was significantly lower (*P* < 0.001, *P* < 0.01) in Zuogui Wan GDM group and control group. The means of fasting blood glucose in high fat model as the control group was lower than that in GDM group, but the difference was not significant. Compared with GDM group, 2hPG was significantly lower (*P* < 0.001) in Zuogui Wan GDM group, control group, and high fat model as the control group.

### 4.4. Biochemical Index

As can be seen from Table 4, compared with GDM group, total cholesterol was significantly lower (*P* < 0.001, *P* < 0.01, and *P* < 0.01) in Zuogui Wan GDM group, control group, and high fat model as the control group. Low density lipoprotein (LDL) was significantly lower (*P* < 0.001, *P* < 0.05) in Zuogui Wan GDM group and control group compared with GDM group. The means of low density lipoprotein in high fat model as the control group was lower than that in GDM group, but the difference was not significant. The means of triglyceride in Zuogui Wan GDM group was lower than that in GDM group, but the difference was not significant. The means of triglyceride in high fat model as the control group was higher than that in GDM group, but the difference was not significant. The means of high density lipoprotein (HDL) in Zuogui Wan GDM group, control group, and high fat model as the control group was lower than that in GDM group, but the difference was not significant.

### 4.5. Insulin, Insulin Resistance Index, Leptin, and Adiponectin

As can be seen from Table 5, compared with GDM group, the insulin was significantly lower (*P* < 0.001, *P* < 0.001) in Zuogui Wan GDM group and control group. Insulin resistance index was significantly lower (*P* < 0.001, *P* < 0.001, and *P* < 0.01) in Zuogui Wan GDM group, control group, and high fat model as the control group than that in GDM group. Compared with GDM group, leptin was significantly lower (*P* < 0.001, *P* < 0.001) in Zuogui Wan GDM group and control group. Compared with GDM group, adiponectin was significantly higher (*P* < 0.05, *P* < 0.001, *P* < 0.001) in Zuogui Wan GDM group, control group, and high fat model as the control group.

## 5. Discussion

Impaired glucose tolerance is a kind of abnormal glucose metabolism status between diabetes and normal glucose tolerance. It is the precursor stage to diabetes with no obvious clinical symptoms. Currently, worldwide, the detection rate for IGT among all regions in both genders is higher than diabetes mellitus [7], according to the statistical materials in China, 8% who suffered from IGT will change to type two diabetes mellitus [8]; intervention work to prevent this is very important.

Our research group has conducted “TCM eugenics project” which has produced abundant results. For instance, it has been found that using traditional Chinese medicine prescriptions to tonify kidney in the embryonic stage can not only promote fetal intrauterine growth and cure intrauterine growth retardation but also improve the immune function of offspring in adult stage significantly [8]. Giving pregnant rats traditional Chinese medicine prescriptions can not only tonify kidney, but also prevent the occurrence of IGT in offspring in adult stage induced by a high fat and sugar diet [9]. These results demonstrate that the “tonifying kidney” method and prescription from traditional Chinese medicine can improve the intrauterine growth environment, inherit excellent biological properties, enhance immunity, and delay or prevent the occurrence of disease in offspring.

Early experiments have demonstrated that giving Zuogui Wan to GDM rats has a therapeutic effect on GDM rats [10]. In this experiment, the birth weight of offspring rats in GDM group was significantly lower than that for other groups. This low weight stemmed from deficiencies during fetal growth. Mother rats from GDM group during pregnancy have obvious symptoms of “three high and one low,” leading to loss of nutrients, so the birth weight of offspring rats was low. But changes occurred as the offspring aged. In the first 6 weeks, body weight in GDM group was lower than that in Zuogui Wan GDM group. But after week 6, GDM group showed an accelerated growth rate and began to catch up with other groups. In the 8th week, the body weight of offspring rats in GDM group had exceeded that in Zuogui Wan GDM group. This result supports Professor Barker's conclusion that “low birth weight children under 2 years old have slow weight gain, but the weight will grow rapidly in later 11 years, which is a fast growing compensatory acquired”; this compensatory production is a risk factor for hypertension, ischemic heart disease, and insulin resistance [11]. Because of administration of Zuogui Wan to their mothers, body weight in Zuogui Wan GDM group did not increase rapidly after week 6, but increased at a steady rate. The results of this study were also consistent with this conclusion from Barker's study.

In week 14, body weight of Zuogui Wan GDM group was significantly lower than that of GDM group. We believe this was because giving Zuogui Wan in pregnancy can strengthen fetal resistance and prevent obesity induced by high fat and sugar diet (especially reducing the abdominal fat weight, the ratio of the abdominal fat weight, and body weight). Insulin resistance is one of the pathological bases for IGT to occur. It has positive association with higher leptin [12] and negative association with adiponectin [13]. In this experiment, the levels of fasting plasma glucose, 2hPG, insulin, and leptin in Zuogui Wan GDM group were significantly lower than in GDM group, but the level of adiponectin was significantly higher than in GDM group. Therefore, it can be concluded that giving Zuogui Wan to GDM rats in embryonic period can reduce the level of fasting blood glucose, 2hPG, insulin, and leptin, fight against insulin resistance and leptin resistance, and also fight against the reduction of adiponectin level induced by high fat and sugar diet. Total cholesterol and LDL in Zuogui Wan GDM group were significantly lower than those in GDM group; triglycerides and HDL in Zuogui Wan GDM group were lower than in GDM group, but the difference was not statistically significant. To some extent, all of these result from treatment in utero.

As can be seen from the experiment result, giving Zuogui Wan to GDM rats in embryonic period can prevent IGT induced by high fat and sugar diet for adult rats. Traditional Chinese medicine believes that patients with diabetes often have some characteristics of “Yang is usually excessive, while Yin is frequently deficient,” which was said by Danxi Zhu, a famous traditional Chinese medicine expert of Yuan Dynasty in China. Maternal gestation period needs blood filling; rats in GDM group must have characteristics of Yin which is often inadequate. The function of Zuogui Wan is nourishing Yin and tonifying the kidney. So it can supply adequate nutrients to the unborn baby rats. This is also consistent with the theory of “supplement the mother, the child will benefit,” which was proposed in an old famous Chinese book named “Jiachuan Nvke Jingyan Zhaiqi.”

In conclusion, the theory “kidney is responsible for reproduction, growth and reproduction,” which has been proposed in Huandi Neijing for more than 2000 years, and whose availability has been proven in its practice, but this theory lacked evidence in modern medical support. This study provides an experimental basis from the perspective of modern medical evidence for the above theory. The result further sheds light on studies focusing on how to protect the offspring's IGT from GDM mothers.

## Figures and Tables

**Table 1:** Body weight (from week 0 to week 15, g).

Group	Week 0	Week 3	Week 6	Week 8	Week 10	Week 12	Week 14	Week 15
GDM group (10)	4.51 ± 0.54	52.27 ± 3.47	138.20 ± 15.43	237.80 ± 26.13	332.60 ± 32.75	360.80 ± 29.25	401.40 ± 26.62	426.20 ± 26.22
Zuogui Wan GDM group (10)	5.11 ± 0.50^*∗*^	58.32 ± 4.31^*∗∗*^	157.80 ± 15.68^*∗∗*^	230.00 ± 34.41	295.90 ± 20.33	329.90 ± 18.38	365.8 ± 19.46^*∗*^	389.80 ± 21.62^*∗*^
Control group (10)	5.29 ± 0.80^*∗∗*^	58.80 ± 3.96^*∗∗*^	149.20 ± 10.09	241.91 ± 11.89	354.90 ± 58.67	391.80 ± 56.25	415.60 ± 57.14	435.90 ± 58.66
High fat model as the control group (10)	5.29 ± 0.80^*∗∗*^	57.43 ± 2.69^*∗∗*^	162.10 ± 12.18^*∗∗∗*^	250.81 ± 24.58	367.60 ± 50.45	402.70 ± 48.59^*∗*^	442.20 ± 49.39^*∗*^	468.60 ± 50.45^*∗*^

Note: all results are presented as mean ± SD. *∗* indicates a significant difference compared with GDM group. ^*∗*^ *P* < 0.05;  ^*∗∗*^ *P* < 0.01; ^*∗∗∗*^ *P* < 0.001.

**Table 2:** Abdominal fat weight/body weight.

Group	Body weight (g)	Abdominal fat weight (g)	Abdominal fat weight/body weight (%)
GDM group (10)	426.20 ± 26.22	23.97 ± 2.88	5.62 ± 0.53
Zuogui Wan GDM group (10)	389.80 ± 21.62^*∗*^	13.54 ± 3.06^*∗∗∗*^	3.49 ± 0.85^*∗∗∗*^
Control group (10)	435.90 ± 58.66	10.84 ± 2.75^*∗∗∗*^	2.56 ± 0.85^*∗∗∗*^
High fat model as the control group (10)	468.60 ± 50.45^*∗*^	17.79 ± 4.85^*∗∗∗*^	3.87 ± 1.15^*∗∗∗*^

Note: all results are presented as mean ± SD. *∗* indicates a significant difference compared with GDM group. ^*∗*^ *P* < 0.05; ^*∗∗∗*^ *P* < 0.001.

**Table 3:** Fasting blood glucose and 2hPG (mmol·L^−1^).

Group	Fasting blood glucose	2hPG
GDM group (10)	5.31 ± 0.28	9.01 ± 0.75
Zuogui Wan GDM group (10)	4.44 ± 0.47^*∗∗∗*^	5.78 ± 0.60^*∗∗∗*^
Control group (10)	4.77 ± 0.64^*∗∗*^	6.50 ± 0.63^*∗∗∗*^
High fat model as the control group (10)	5.02 ± 0.32	6.77 ± 0.70^*∗∗∗*^

Note: all results are presented as mean ± SD. *∗* indicates a significant difference compared with GDM group. ^*∗∗*^ *P* < 0.01; ^*∗∗∗*^ *P* < 0.001.

**Table 4:** Biochemical Index (mmol·L^−1^).

Group	Total cholesterol	Triglyceride	Low density lipoprotein	High density lipoprotein
GDM group (10)	2.86 ± 0.44	0.56 ± 0.09	0.62 ± 0.08	0.51 ± 0.08
Zuogui Wan GDM group (10)	2.15 ± 0.27^*∗∗∗*^	0.46 ± 0.10	0.38 ± 0.08^*∗∗∗*^	0.44 ± 0.10
Control group (9)	2.34 ± 0.43^*∗∗*^	0.56 ± 0.11	0.51 ± 0.11^*∗*^	0.47 ± 0.07
High fat model as the control group (10)	2.35 ± 0.16^*∗∗*^	0.63 ± 0.20	0.55 ± 0.06	0.48 ± 0.05

Note: all results are presented as mean ± SD. *∗* indicates a significant difference compared with GDM group. ^*∗*^ *P* < 0.05; ^*∗∗*^ *P* < 0.01; ^*∗∗∗*^ *P* < 0.001.

**Table 5:** Insulin, insulin resistance index, leptin, and adiponectin.

Group	Insulin (mLU·L^−1^)	HOMA-IR (mmol*∗*mLU·L^−2^)	Leptin (ng·L^−1^)	Adiponectin (*μ*g·mL^−1^)
GDM group (10)	10.27 ± 0.34	2.45 ± 0.16	5.61 ± 0.06	63.17 ± 1.51
Zuogui Wan GDM group (10)	9.00 ± 0.15^*∗∗∗*^	1.77 ± 0.17^*∗∗∗*^	4.57 ± 0.07^*∗∗∗*^	68.40 ± 4.40^*∗*^
Control group (9)	8.33 ± 0.49^*∗∗∗*^	1.87 ± 0.14^*∗∗∗*^	4.49 ± 0.12^*∗∗∗*^	81.09 ± 3.27^*∗∗∗*^
High fat model as the control group (10)	10.28 ± 0.30	2.18 ± 0.31^*∗∗*^	5.88 ± 0.25	74.89 ± 5.10^*∗∗∗*^

Note: all results are presented as mean ± SD. *∗* indicates a significant difference compared with GDM group. ^*∗*^ *P* < 0.05; ^*∗∗*^ *P* < 0.01; ^*∗∗∗*^ *P* < 0.001.
